# Reply to Sah et al.

**DOI:** 10.1055/a-2777-8310

**Published:** 2026-01-27

**Authors:** Hiroki Fukuya, Eikichi Ihara, Yoichiro Iboshi, Yorinobu Sumida, Daisuke Yoshimura, Shohei Hamada, Taisuke Sasaki, Akito Ohkubo, Shuichi Itonaga, Hitoshi Homma, Ryota Okitsu, Akihisa Ohno, Mitsuru Esaki, Naohiko Harada

**Affiliations:** 1 Department of Gastroenterology, National Hospital Organisation Kyushu Medical Center, Fukuoka, Japan; 2 Institute of Research Center, National Hospital Organization Kyushu Medical Center, Fukuoka, Japan; 3 12923 Department of Medicine and Bioregulatory Science, Graduate School of Medical Sciences, Kyushu University, Fukuoka, Japan; 4 Department of Gastroenterology, Kitakyushu Municipal Medical Center, Fukuoka, Japan



We sincerely thank Drs. Sah and Kumbhalwar for their thoughtful letter and interest in our study 1 . We appreciate their recognition of our approach to capturing post-endoscopic submucosal dissection (ESD) burden across both minor and major post-procedural events.

Regarding residual confounding, we acknowledge that both the age-adjusted Charlson Comorbidity Index (ACCI) and the American Society of Anesthesiologists Physical Status (ASA-PS) classification reflect important aspects of patients' overall condition. However, in our propensity-score model, we deliberately selected ACCI as the primary comorbidity measure because ACCI and ASA-PS represent conceptually distinct constructs that cannot realistically be balanced simultaneously 2 . Enforcing tight balance on both indices would have substantially reduced the number of matched pairs. ACCI incorporates both age and a comprehensive range of comorbidities, and our matching achieved an absolute standardized difference of 0.10, indicating satisfactory balance. While some residual confounding may persist, we believe this represents a reasonable compromise for an exploratory study.

Concerning exposure characterization, we agree that our dichotomous classification of glucocorticoid/immunomodulator (GC/IM) use represents a simplification 3 . Although exploratory subgroup analyses by agent type and GC dose revealed no clear patterns, these analyses were constrained by small sample sizes, especially in the IM-only subgroup. In addition, because of the inherent limitations of a retrospective study design, we were unable to access complete information on cumulative dose, treatment duration, or other treatment-specific variables for all patients. Future studies with prospective data collection will be essential to strengthen the biological plausibility framework and better inform counseling for patients receiving different immunosuppressive regimens.

The concern regarding our composite endpoint is valid. As an exploratory study in a field lacking established evidence, we selected a pragmatic composite endpoint to capture overall post-ESD clinical workload. Minor symptoms such as pain 4 or low-grade fever 5 frequently necessitate bedside assessments, medication administration, and occasionally imaging, thereby contributing to real-world resource utilization. Concurrently, we analyzed major complications—delayed bleeding and perforation—separately as clinically critical outcomes; these remained uncommon and showed no between-group differences. While more stratified burden analyses (e.g., symptom-specific lengths of stay or readmission risk) would provide additional insight, our limited sample size constrained the robustness of such modeling.

Finally, we appreciate the emphasis on the febrile patient subgroup. The overall absence of significant differences in hospitalization duration appears largely attributable to the very small number of febrile cases and the fact that patients with pain alone generally achieved adequate symptom control with analgesics, allowing timely discharge. Although the fever subgroup demonstrated a numerically longer median hospital stay, the sample size was insufficient to determine clinical significance. Only a larger dataset could clarify whether early fever truly signals prolonged recovery risk in GC/IM users undergoing gastric ESD and help refine triage and follow-up strategies.

We appreciate the constructive comments by Drs. Sah and Kumbhalwar, which highlight important methodological and clinical issues for future research. We hope that our study, together with their letter, will contribute to more nuanced risk stratification, informed consent, and postoperative care for immunosuppressed patients undergoing gastric ESD.

**Publication note**

Letters to the editor do not necessarily represent the opinion of the editor or publisher. The editor and publisher reserve the right to not publish letters to the editor, or to publish them abbreviated or in extracts.
